# Cholic acid for primary bile acid synthesis defects: a life-saving therapy allowing a favorable outcome in adulthood

**DOI:** 10.1186/s13023-018-0920-5

**Published:** 2018-10-29

**Authors:** Emmanuel Gonzales, Lorenza Matarazzo, Stéphanie Franchi-Abella, Alain Dabadie, Joseph Cohen, Dalila Habes, Sophie Hillaire, Catherine Guettier, Anne-Marie Taburet, Anne Myara, Emmanuel Jacquemin

**Affiliations:** 1 Pediatric Hepatology and Pediatric Liver Transplantation Unit, National Reference Centre for rare pediatric liver diseases and Filfoie, Hôpital Bicêtre, Assistance Publique-Hôpitaux de Paris, Paris, France; 2 Faculty of Medicine Paris - Sud, University Paris – Sud / Paris - Saclay, Paris, France; 3 INSERM UMR-S1174 and Hepatinov, University Paris –Sud / Paris – Saclay, Orsay, France; 4 University of Trieste, Trieste, Italy; 5 Pediatric Radiology Unit, Hôpital Bicêtre, Assistance Publique - Hôpitaux de Paris, Paris, France; 6 Department of Pediatrics, Hôpital Sud, Rennes, France; 7 Hepatology Unit, Hôpital Foch, and Hôpital Beaujon, Assistance Publique - Hôpitaux de Paris, Paris, France; 8 Pathology Unit, Hôpital Bicêtre, Assistance Publique - Hôpitaux de Paris, Paris, France; 9 Pharmacy Unit, Hôpital Bicêtre, Assistance Publique - Hôpitaux de Paris, Paris, France; 10 Groupe Hospitalier Paris Saint-Joseph, Paris, France; 11 Service d’Hépatologie et de Transplantation Hépatique Pédiatriques, Hôpital Bicêtre, 78, rue du Général Leclerc, Le Kremlin-Bicêtre, France

**Keywords:** Bile acid, Genetic cholestasis, *AKR1D1*, *HSD3B7*

## Abstract

**Background:**

Oral cholic acid (CA) replacement has been shown to be an effective therapy in children with primary bile acid synthesis defects, which are rare and severe genetic liver diseases. To date there has been no report of the effects of this therapy in children reaching adulthood. The aim of the study was to evaluate the long-term effectiveness and safety of CA therapy.

**Methods:**

Fifteen patients with either 3β-hydroxy-Δ^5^-C_27_-steroid oxidoreductase (3β-HSD) (*n* = 13) or Δ^4^–3-oxosteroid 5β-reductase (Δ^4^–3-oxo-R) (*n* = 2) deficiency confirmed by mass spectrometry and gene sequencing received oral CA and were followed prospectively.

**Results:**

The median age at last follow-up and the median time of follow-up with treatment were 24.3 years (range: 15.3–37.2) and 21.4 years (range: 14.6–24.1), respectively. At last evaluation, physical examination findings and blood laboratory test results were normal in all patients. Liver sonograms were normal in most patients. Mean daily CA dose was 6.9 mg/kg of body weight. Mass spectrometry analysis of urine showed that excretion of the atypical metabolites remained low or traces in amount with CA therapy. Liver fibrosis scored in liver biopsies or assessed by elastography in 14 patients, after 10 to 24 years with CA therapy, showed a marked improvement with disappearance of cirrhosis (median score < F1; range: F0-F2). CA was well tolerated in all patients, including five women having 10 uneventful pregnancies during treatment.

**Conclusions:**

Oral CA therapy is a safe and effective long-term treatment of 3β-HSD and Δ^4^–3-oxo-R deficiencies and allows affected children to reach adulthood in good health condition without the need for a liver transplantation.

## Background

Inborn errors of primary bile acid synthesis are rare inherited autosomal recessive disorders. The most frequent defects are the 3β-Δ^5^-hydroxy-C_27_-steroid oxidoreductase (3β-HSD) deficiency (OMIM 607765) which is due to mutations in *HSD3B7*; and to a lesser extent the Δ^4^–3-oxosteroid-5β-reductase (Δ^4^–3-oxo-R) deficiency (OMIM 235555) due to mutations in *AKR1D1* [1–7]. These defects in enzymes catalyzing key reactions in the formation of the primary bile acids (BA), namely cholic acid (CA) and chenodeoxycholic acid (CDCA) in human, lead to an inadequate synthesis of primary BA that are critical for bile formation and to the production and the accumulation of atypical and hepatotoxic BA intermediates [8–11]. These deficiencies are most commonly manifest in neonates or infants as cholestasis and can progress to early cirrhosis and liver failure unless treated [1, 2, 7, 9, 10]. Absence of pruritus, normal serum γ-glutamyltransferase (GGT) activity, and normal or low total serum bile acid concentration are diagnostic features of these conditions [12]. Specific diagnosis is based on mass spectrometry (MS) analysis of urinary bile acids showing typical bile acid profiles and on the identification of disease-causing mutations in *HSD3B7* or in *AKR1D1* [1–10]. Oral primary bile acid replacement by CA or CDCA is required for these defects in order to restore bile flow and to down-regulate endogenous bile acid synthesis [9, 10, 13–16]. CA is the major primary bile acid in humans and is now recognized as the bile acid of choice since it is neither hepatotoxic nor embryotoxic/teratogenic, and is effective therapy for 3β-HSD and Δ^4^–3-oxo-R deficiencies [9, 10, 14, 16–19]. CA is the only primary BA having a marketing authorization in the USA [20] and in Europe [21] in these two indications. Prolonged oral CA therapy is considered safe and lifesaving during childhood as it leads to normalization of clinical features, serum liver biochemistry and liver imaging, together with a substantial improvement of mass spectrometry bile acid profiles and liver histology [14, 16–19]. However, published data concerning the long-term outcome of such patients treated with oral CA and reaching adulthood are lacking. In this study, we updated the follow-up, after around 20 years of CA therapy, of a previously reported cohort of 15 patients with 3β-HSD or Δ^4^–3-oxo-R deficiencies [14], most of whom having now reached adulthood. The data confirm the favorable outcome with treatment, mainly the decrease in liver fibrosis, and show that CA therapy should guarantee a normal quality of life during adulthood.

## Methods

### Patients and study design

the 15 patients (13 with 3β-HSD deficiency [patients 1–13, families A-I] and two with Δ4–3-oxo-R deficiency [patients 14, 15, family J]), prospectively followed from 1993 to August 2007 and reported in 2009 [14] were again followed prospectively during CA therapy from August 2007 to 2017 (Table 1). This new 10-year period of time of follow-up analysis represents the basis of the study. As previously described, the follow-up evaluations were performed every year and included: 1) physical examination at least once a year; 2) blood liver biochemistry tests including alpha-fetoprotein and total serum bile acids; 3) abdominal ultrasonography; 4) urine bile acid analyses by gas chromatography/mass spectrometry (GC-MS). Bile acid analyses in urine samples were performed as previously described [14] and predominant specific atypical bile acids were determined in urine and expressed as a percentage of total urinary bile acids (physiological plus atypical, in μmol/mmol creatinine). In the 3β-HSD deficiency, 3β-hydroxylated-Δ^5^ derivatives were assayed whereas in Δ^4^–3-oxo-R deficiency, 3-oxo-Δ^4^ derivatives were assayed. In addition, liver biopsy and/or liver elastography (Transient eslastography (Fibroscan) (TE), supersonic shear imaging (SSI), or acoustic radiation force impulse (ARFI) technologies) were performed when indicated and according to patient availability. Liver biopsy specimens were analyzed by the same pathologist (C. G.) and compared to previous biopsies. Portal fibrosis and activity were scored according to the grading system of Metavir, and severity and localization of cholestasis were assessed as previously described for these patients [14]. Data of the liver elastography measurements with TE, SSI, or ARFI technologies were used to estimate liver fibrosis. Because none of these methods have been validated in hereditary defects of primary bile acid synthesis, we chose cut-off values for fibrosis staging (F0-F4) previously reported in chronic liver diseases: 1) The cut off values of TE [22] were: F1 ≥ 6.5, F2 ≥ 8.1, F3 ≥ 10.8, F4 ≥ 13.4; 2) The cut off values of SSI [23] were: F1 ≥ 7.5, F2 ≥ 8.04, F3 ≥ 9.27, F4 ≥ 11.12. For ARFI, a value < 1.207 m/s was considered normal (F0) [24]. Quality of life, social status and professional activity of the patients were assessed by history taking at each clinic visit in our centre.

**Table 1:** Characteristics of 15 patients diagnosed with either a 3β-hydroxy-Δ^5^-C_27_-steroid oxidoreductase deficiency or a Δ^4^–3-oxosteroid 5β-reductase deficiency and who received long-term therapy with cholic acid

Patient^a^ (Family)	Sex	Age at CA therapy initiation (years)	Duration of CA therapy (years)	Age at last follow-up (years)	Daily dose of CA at last follow-up	
					mg.kg^−1^	mg
*3β-HSD deficiency*						
1 (A)	M	3.9	24.1	28.0	6.1	500
2 (B)	F	4.3	24.1	28.4	6.9	450
3 (C)	F	7.8	24.1	31.8	5.8	350
4 (D1)	F	4.3	24.1	28.3	7.5	400
5 (D2)	F	0.6	14.6	15.3	6.5	300
6 (E1)	M	5.2	24.1	29.3	6.4	500
7 (E2)	F	13.1	24,1	37.2	8.3	500
8 (F)	M	2.3	21.9	24.3	8.4	400
9 (G1)	F	2.3	21.4	23.8	8.3	450
10 (G2)	F	11.6	21,4	33.1	9.6	500
11 (H)	F	0.8	20.1	20.9	5.5	300
12 (I1)	M	5.1	16.2	21.3	4.8	350
13 (I2)	F	0.3	15.5	15.8	3.4	300
*Δ* ^*4*^ *–3-oxo-R deficiency*						
14 (J1)	F	0.7	20.0	20.8	7.8	400
15 (J2)	F	0.7	20.0	20.8	7.5	400
Median		3.9	21.4	24.3	6.9	400
Range		0.3–13.1	14.6–24.1	15.3–37.2	3.4–9.6	300–500

^a^, The numbering of the patients refers to a previous reference reporting in details on demographics and genetic characteristics and on the initial symptoms of the patients [14]. 3β-HSD, 3β-hydroxy-Δ5-C27-steroid oxidoreductase; Δ4–3-oxo-R, Δ4–3-oxosteroid 5β-reductase; CA, cholic acid

### Treatment

From 1993 to August 2007, patients received cholic acid as previously reported [14]. Since August 2007, patients received cholic acid (Orphacol, Laboratoires CTRS, Boulogne-Billancourt, France) in two divided doses with 50 mg and/or 250 mg capsules [21]. At this date, the mean daily dose of CA was 6.3 mg/kg and 5.3 mg/kg in 3β-HSD deficiency and Δ^4^–3-oxo-R deficiency patients, respectively [14]. One patient with Δ^4^–3oxo-R deficiency (patient 14) was still receiving daily 4 mg/kg of UDCA (200 mg/d) given separately from CA. This small amount of UDCA was necessary in this patient to maintain normal serum liver biochemistry tests, which were otherwise slightly abnormal. Doses of CA were thereafter adapted individually based upon the results of the follow-up urinary bile acid analyses by GC-MS and serum liver tests. The dosage of 500 mg/day, corresponding to the physiologic daily synthesis rate for primary bile acids in healthy adults [8], was considered as a maximum.

## Results

The median age at last follow-up and the median time of follow-up with treatment were 24.3 years (range: 15.3–37.2) and 21.4 years (range: 14.6–24.1), respectively (Table 1). At last evaluation, physical examination findings and blood laboratory test (liver tests, alphafetoprotein, prothrombin time, factor V, albumin, bile acids, and fat-soluble vitamins) were normal in all patients. Growth in weight and height were within the normal range in all patients. Areflexia or decreased tendon reflexes observed after 5 years with CA therapy in patients 2, 3, 6 and 7 was still observed in these four patients at last follow-up. Liver sonograms were normal in most patients. However, hyperechogenic liver and dystrophic liver were observed in 2 patients (patients 6, 11) and 1 patient (patient 14), respectively. None of the 15 patients had ultrasonographic evidence for liver dysplasia. Gallstones were observed in 1 patient (patient 3). Of note, 2 patients (patients 1 and 4) underwent previously a cholecystectomy for a gallstone disease which did not recur after surgery. Renal cysts initially present in five children with 3β-HSD deficiency and no longer detectable after few years of CA therapy remained undetectable at last follow-up. Mean daily CA dose was 6.9 mg/kg of body weight. Patient 14 still receives UDCA (200 mg/d; 3 mg/kg/d). Mass spectrometry analysis of urinary bile acids showed that excretion of the atypical metabolites remained low or traces in amount with CA therapy. In the 13 patients with 3β-HSD deficiency, at initial evaluation and before CA therapy, 3β-hydroxylated-Δ^5^ derivatives represented an average of 94% of total urinary bile acids. These atypical bile acids were dramatically reduced at last evaluation in our previous report after a median follow-up of around 10 years with CA therapy [14]. In the present report and at last evaluation after a median follow-up of 21.9 years with CA therapy, these atypical metabolites were still dramatically reduced by 300 fold (mean: 0.064 μmol/mmol creatinine) and were undetectable in six patients (patients 1, 2, 7, 11, 12. 13). However, in case of imperfect compliance (patients 6 and 8), we observed an increase in the amount of these atypical BA, which decreased again to their usual levels after improvement of compliance (patient 6). In the two patients with Δ^4^–3-oxo-R deficiency, at initial evaluation and before CA therapy, 3-oxo-Δ^4^ derivatives represented an average of 89% of total urinary bile acids. These atypical bile acids were highly reduced by 30 fold at last evaluation in our previous report after a follow-up of around 10 years with CA therapy [14]. In the present report and at last evaluation after a follow-up of 20 years with CA therapy, these atypical bile acids remained at similar levels with a reduction of 37 fold (1.81 μmol/mmol creatinine). No problem of compliance was observed in these 2 patients.

**Fig. 1:**
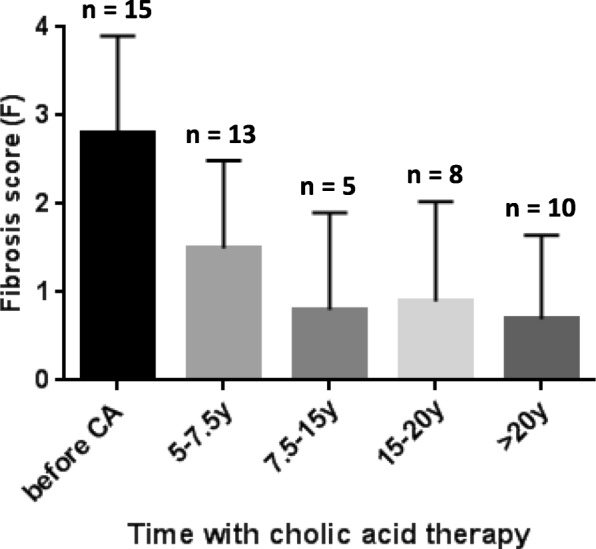
Effect of long-term oral CA therapy on liver fibrosis in 15 children with 3β-hydroxy-Δ5-C27-steroid oxidoreductase deficiency (*n* = 13) or Δ4–3-oxosteroid 5β-reductase deficiency (*n* = 2). Fibrosis scores were assessed according to Metavir in liver biopsy specimens or as described in the patients and methods section in cases of assessment using non-invasive methods. See Table 2 for the details of fibrosis scores. Data are presented as mean +/− SD values. When in a patient 2 values of fibrosis score were recorded during the same time period, only the most recent score was considered to calculate the mean +/− SD values

**Fig. 2:**
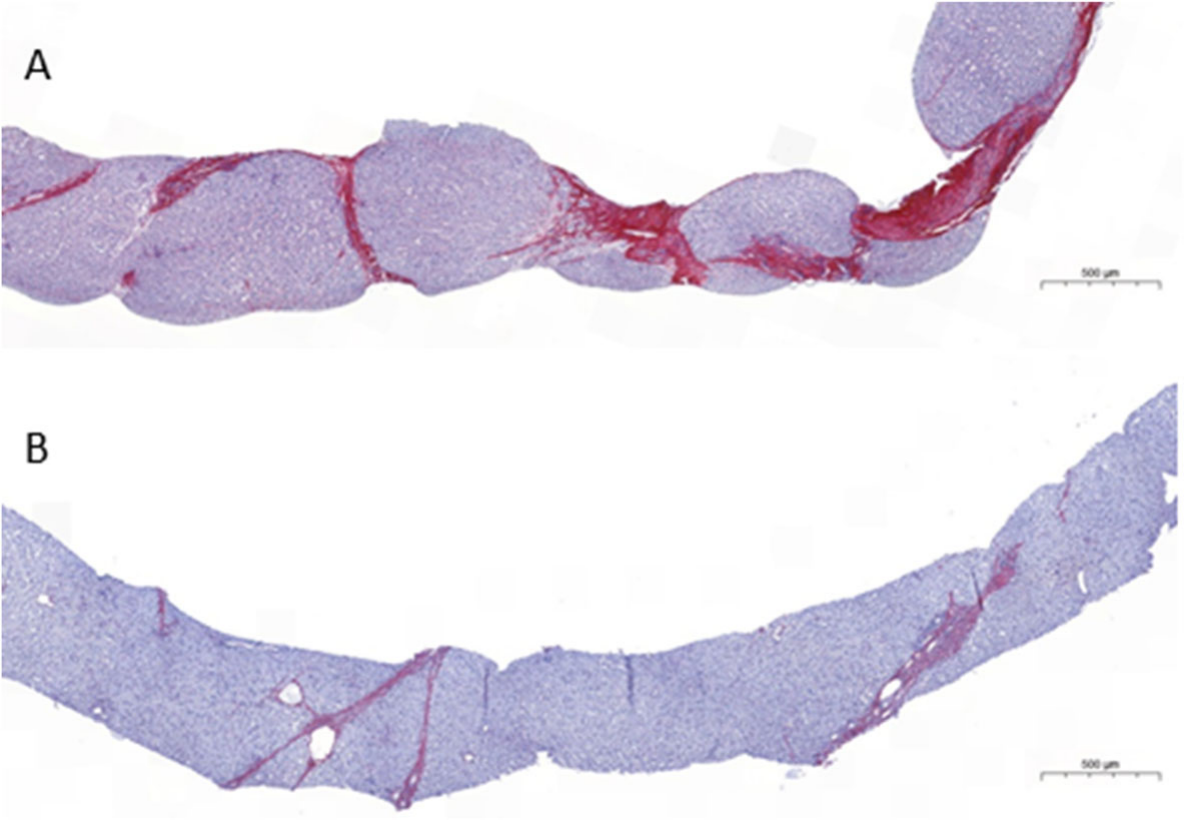
Initial liver biopsy specimens and the effect of long-term oral CA therapy in patient 10 with 3β-hydroxy-Δ5-C27-steroid oxidoreductase deficiency. **a** Liver histology at onset of CA therapy. **b** Liver histology after 11.5 years of CA therapy. See Table 2 for fibrosis scores

**Fig. 3:**
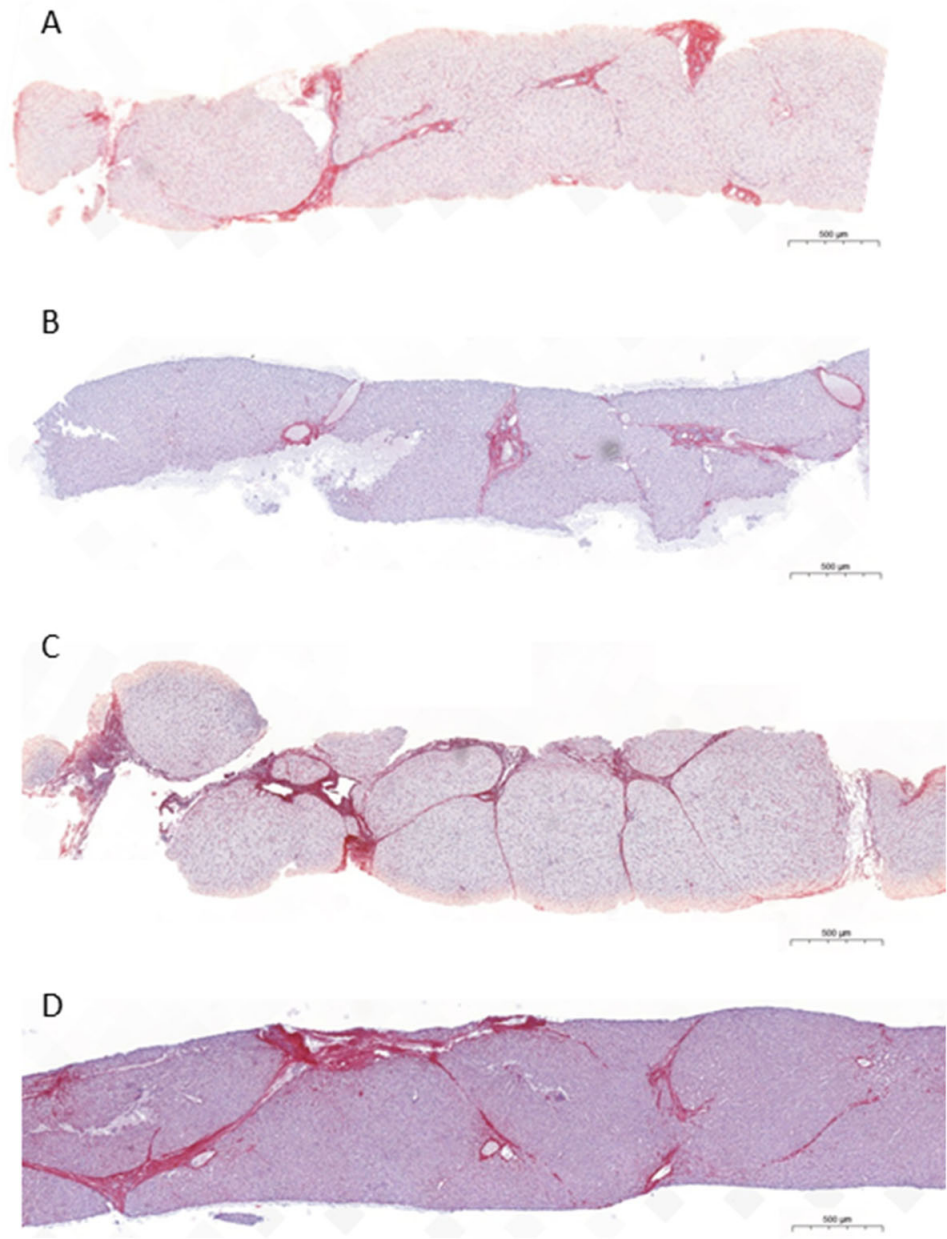
Evolution during long-term CA therapy of liver pathology in patient 15 (**a** and **b**) and patient 14(**c** and **d**) with Δ4–3-oxosteroid 5β-reductase deficiency. **a** Liver histology after 5.5 years of combined UDCA plus CA therapy. **b** Liver histology after 16 years of combined UDCA plus CA therapy. **c** Liver histology after 5.5 years of CA therapy. **d** Liver histology after 16 years of CA therapy. See Table 2 for fibrosis scores

Liver fibrosis scored in liver biopsies (*n* = 3, patients 10, 14 and 15) or assessed by elastography (*n* = 23 measurements, all patients but patient 2), after 7.5 to more than 20 years of CA therapy, showed a marked improvement with disappearance of cirrhosis (patients 4, 7, 14) (Table 2). The evolution over time of the mean score of fibrosis in the 15 patients is presented in Fig. 1. In the only patient in whom liver fibrosis was not evaluated after 10 years with CA therapy, liver sonogram was normal at age 27.9 and after 23.6 years with CA (patient 2, family B). No signs of cholestasis and of inflammatory activity were seen in the biopsy of patient 10 (family G) with 3β-HSD deficiency performed after 11.5 years of treatment (Fig. 2) and in the two biopsies performed after 16 years of treatment in patients 14 and 15 (Family J) with Δ^4^–3-oxo-R deficiency (Fig. 3). No patients developed portal hypertension. CA was well tolerated in all patients. Sexual maturation progressed normally in all patients (female and male), including in the two girls who did not yet reach adult age (patients 5 and 13). While treated with CA, four women with 3β-HSD deficiency (patients 2, 7, 9, 10) and one woman with Δ^4^–3-oxo-R deficiency (patient 14) have had nine uneventful pregnancies and one uneventful pregnancy, respectively. During all pregnancies doses of CA were not modified and all newborns were healthy.

**Table 2:** Fibrosis score before and after cholic acid therapy in 15 patients diagnosed with either a 3β-hydroxy-Δ5-C27-steroid oxidoreductase deficiency (patients 1–13) or a Δ4–3-oxosteroid 5β-reductase deficiency (patients 14–15)

Patient (Family)	Fibrosis score ^a^,^b^						
	before CA	5–7.5 years with CA	7.5 - < 15 years with CA	15 - < 20 years with CA		≥20 years with CA	
1 (A)	F2^a^	F1^a^ 5.1 y				F0^b^ TE = 5.4 kPa 22 y	
2 (B)	F3^a^	F2^a^ 5.4 y					
3 (C)	F3^a^	F0^a^ 6.5 y				F0^b^ ARFI = 1 m/s 22 y	
4 (D1)	F4^a^	F3/F2^a^ 7.2 y	F2^b^ TE = 8.3 kPa 14.7 y			F2^b^ TE = 10 kPa 22 y	
5 (D2)	F1^a^		F0^b^ TE = 3.3 kPa 13 y				
6 (E1)	F3^a^	F1^a^ 5.2 y		F1^b^ TE = 6.7 kPa 17 y		F3^b, £,+^ TE = 13 kPa 21 y	F1^b^ TE = 7.8 kPa 24 y
7 (E2)	F4^a^	F2^a^ 5.3 y				F0^b^ TE = 4.8 kPa 21 y	
8 (F)	F3^a^	F1^a^ 5.3 y				F2^b,+^ SSI = 8.1 kPa (6.8–9.4) 20 y	F2^b,+^ SSI = 8.5 kPa (7.1–9.3) 21 y
9 (G1)	F3^a^	F2^a^ 5.6 y		F0^b^ TE = 4.4 kPa 19 y		F0* SSI = 4.9 kPa (4.4–6.1) 21 y	
10 (G2)	F4/F3^a^		F2^a^ 11.5 y	F2^b^ TE = 8.2 kPa 18y		F2^b^ SSI = 8.2 kPa (7.9–8.5) 21 y	
11 (H)	F3^a^	F1^a^ 5.3 y		F0^b^ SSI = 5.4 kPa (4.4–6.9) 19 y			
12 (I1)	F3^a^	F0^a^ 6.1 y	F0^b^ TE = 4.6 kPa 13 y	F1^b^ SSI = 7.7 kPa (6–9) 15.1 y			
13 (I2)	F0^a^	F1^a^ 5.5 y	F0^b^ TE = 4.3 kPa 12 y	F0^b^ SSI = 6.7 kPa (5.6–7.7) 15 y			
14 (J1)	F4^a^	F4/F3^a^ 5.5y		F3^a^ 16 y		F0^b^ SSI = 7.3 kPa (5.7–9.6) 20y	
15 (J2)	F3^a^	F2^a^ 5.5y		F1/F2^a^ 16y	F0^b^ SSI = 6.4 kPa (5.4–7.8) 18y	F0^b^ SSI = 6.9 kPa (6.5–7.6) 20y	
Median score	F3	F1	F0	F0.5		F0	
Range	F0-F4	F0-F4/F3	F0-F2	F0-F3		F0-F2	

Liver fibrosis was assessed according to Metavir score in liver biopsy specimens ^a^, or using the following non invasive methods^b^: TE, transient elastography; SSI, supersonic shear imaging; or ARFI, acoustic radiation force impulse. The duration of cholic acid therapy at the time of the assessment of liver fibrosis is indicated below each result. £, chronic alcohol intake; +, poor compliance to cholic acid therapy. When in a patient 2 values of fibrosis score were recorded during the same time period, only the more recent value was considered to calculate the median and range values. See patients and methods section for details. CA, cholic acid

At the time of data collection, 5 patients were still students (2 at school (college), 3 at university), 8 were employed (1 is a nurse, 1 is a teacher, 1 is an assistant in a pet store, 1 is a garbage collector, 1 is a sports educator, 1 is a qualified worker, 2 are engineers) and 2 were housewives. All the patients reported a normal quality of life.

## Discussion

Oral bile acid therapy has been reported to be effective in the treatment of the most common primary bile acid synthesis defects and CA therapy is the recommended therapy in these rare diseases, which are commonly lethal or require liver transplantation if untreated. The aim of this study was to make an update of the follow-up after around 20 years of CA therapy of our previously reported cohort of 15 patients with 3β-HSD or Δ^4^–3-oxo-R deficiencies [14], most of them having now reached adulthood, and to evaluate the long-term effectiveness and safety of CA therapy. The data confirm the favorable outcome with treatment, mainly the decrease in liver fibrosis, and show that CA therapy is safe and should guarantee a normal quality of life during adulthood.

The 15 children with a genetic defect in primary bile acid synthesis received oral CA treatment for a median period of 21.4 years. With CA therapy, the long-term outcome and the biochemical control of the patients were excellent. All patients are alive with their native liver, with normal findings on physical examination, unless hypo/areflexia in 4 patients being the irreversible consequence of prolonged vitamin E deficiency before disease diagnosis. Also, all patients had normal serum liver biochemistry tests and the excretion of the atypical metabolites of bile acids remained low or traces in amount, signing a good metabolic control of the primary bile acid synthesis defects with CA therapy. However, for patients with 3β-HSD deficiency, one study suggested that tandem mass spectrometry could allow a more accurate quantification of 3β-hydroxylated-Δ^5^ derivatives [25]. Most patients had normal liver ultrasonography and 4 patients had minor ultrasonographic liver abnormalities. In all patients serum alphafetoprotein level was persistently normal and none of the patients developed hepatocellular carcinoma. Liver fibrosis scored in liver biopsies and/or by elastography in 14 patients, after 10 to 24 years of CA therapy, seems to confirm in the long-term the previously reported marked improvement of fibrosis with time compared to initial scores. Of note we observed a disappearance of cirrhosis in 3 patients, as it has been reported in other liver diseases [26]. Among the non-invasive methods of measuring liver stiffness that is supposed to mainly result from fibrosis, TE, SSI and ARFI technologies have shown good accuracy for the detection and quantification of liver fibrosis in adult series [22–24, 27]. These series concerned patients with different types of liver diseases, including patients with chronic cholestasis but no patients with cholestasis due to a primary defect in bile acid synthesis were reported in these series. Therefore, the absence of previous validation of these technologies to evaluate liver fibrosis in the patients reported here may represent a limitation in the interpretation of the data. Only two patients had a worsening of fibrosis score after 20 years of therapy (patients 6 (E1) and 8 (F)). In patient 6, this was transient and likely due to chronic alcohol intake and poor compliance to therapy since the age of 18 years; fibrosis score improved after good compliance and alcoholic withdrawal were achieved. In patient 8, worsening of fibrosis score can be explained by a chronic imperfect compliance.

The study confirmed the safety of CA therapy, including during pregnancy. No serious adverse events were observed with a cumulative duration of treatment of more than 300 patient-years and five women treated at therapeutic/physiologic doses have given birth to 10 healthy newborns. This shows that CA is unlikely to be embryotoxic when taken at therapeutic (physiologic) doses during pregnancy. Last but not least, all the patients had a social status and a professional activity similar to the general population.

## Conclusions

All in all, these data show that oral CA therapy is a safe and effective long-term treatment of the most common primary bile acid synthesis defects. CA therapy allows affected children to reach adulthood in good health condition without the need for a liver transplantation. Provided a successful transition and a follow-up during adulthood performed by a specialized hepatologist, it is likely that CA therapy will guarantee to patients, including patients diagnosed at adult age, a normal quality of life throughout all their lifetime [28–30].

## Abbreviations

3β-HSD: 3β-hydroxy-Δ5-C27-steroid oxidoreductase
ALT: Alanine aminotransferase
ARFI: Acoustic radiation force impulse
CA: Cholic acid
CDCA: Chenodeoxycholic acid
DCA: Deoxycholic acid
FAB-MS: Fast atom bombardment/mass spectrometry
GC-MS: Gas chromatography/mass spectrometry
GGT: Gamma-glutamyl transferase
SSI: Supersonic shear imaging
TE: Transient elastography
UDCA: Ursodeoxycholic acid
Δ4–3-oxo-R: Δ4–3-oxosteroid 5β-reductase
